# Temperature effects on the C–H symmetric stretching vibrational frequencies of guest hydrocarbon molecules in 5^12^, 5^12^6^2^ and 5^12^6^4^ cages of sI and sII clathrate hydrates†

**DOI:** 10.1039/d0ra06668k

**Published:** 2020-10-12

**Authors:** Go Fuseya, Satoshi Takeya, Akihiro Hachikubo

**Affiliations:** Kitami Institute of Technology 165, Koen-cho Kitami 090-8507 Japan hachi@mail.kitami-it.ac.jp; National Institute of Advanced Industrial Science and Technology (AIST) Central 5, 1-1-1, Higashi Tsukuba 305-8565 Japan

## Abstract

C–H symmetric stretching vibrational frequencies of CH_4_, C_2_H_4_ and C_2_H_6_ molecules encapsulated in 5^12^, 5^12^6^2^ and 5^12^6^4^ cages of structures I (sI) and II (sII) clathrate hydrates measured by Raman spectroscopy in the temperature range of 93–183 K was analysed. The slopes of the symmetric stretch vibrational frequencies under changing temperatures (Δ*v*/Δ*T*) for CH_4_, C_2_H_4_ and C_2_H_6_ molecules encapsulated in sII 5^12^6^4^ cages were smaller than those for molecules in sI 5^12^6^2^ cages, although sI 5^12^6^2^ cages are smaller than sII 5^12^6^4^ cages. We compared the results of Δ*v*/Δ*T* in this study with the geometrical properties of each host water cage, and these comparisons suggest that the geometry of host water cages affects Δ*v*/Δ*T*.

## Abstract 2

Temperature effects on the C–H symmetric stretch of hydrocarbons in various cages of sI and sII clathrate hydrates were observed.

## Graphical abstract

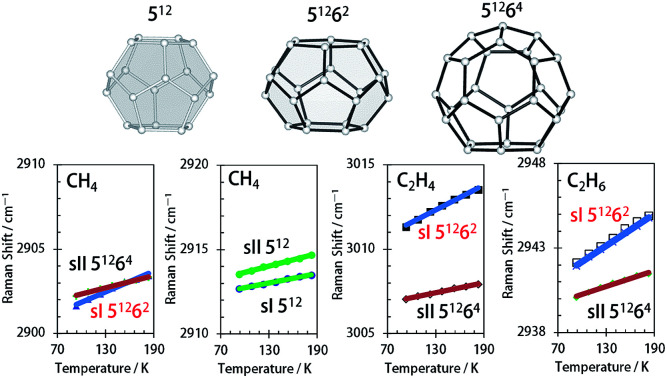

## Introduction

Clathrate hydrates, commonly known as gas hydrates, are crystalline inclusion compounds consisting of guest molecules of suitable sizes and shapes encapsulated in well-defined cages formed by water molecules. Gas hydrates with encapsulated hydrocarbon gases, which exist in sea/lake bottom sediments, have attracted considerable interest as a potential source of natural gas.^1–3^ There are three common crystallographic structures of hydrates, structure I (sI), structure II (sII) and structure H (sH).^4–6^ The unit cell of sI hydrates comprises two pentagonal dodecahedral (5^12^) and six tetrakaidecahedral (5^12^6^2^) water cages.^4^ For sII hydrates, the unit cell is formed by sixteen 5^12^ cages and eight hexakaidecahedral (5^12^6^4^) water cages.^5^ Small guest molecules such as methane (CH_4_), ethylene (C_2_H_4_) and ethane (C_2_H_6_) form sI hydrates, whereas larger molecules such as propane (C_3_H_8_) or 2-methylpropane (C_4_H_10_) form sII hydrates. C_2_H_4_ and C_2_H_6_ are encapsulated in sI 5^12^6^2^ cages, while sI 5^12^ cages remain almost empty at equilibrium pressure conditions except at high-pressure conditions (∼100 MPa).^7–10^ A mixture of CH_4_ + C_2_H_6_ hydrates can form both sI and sII hydrates, depending on the gas composition of CH_4_ and C_2_H_6_.^11^ Coexisting states of both sI and sII CH_4_ + C_2_H_6_ hydrates have also been observed in natural settings.^12^ Moreover, in environments where larger hydrocarbons, *e.g.*, C_3_H_8_ or C_4_H_10_ are present, smaller hydrocarbon molecules can also be encapsulated in the sII hydrate.^13^ Thus, accumulating knowledge about hydrocarbon hydrates in sI and sII structures is important.

Raman spectroscopy has been frequently used for gas hydrates to identify the type of crystal structure^11,14,15^ or to analyse cage occupancy^14^ and type of guest molecule.^15^ The Raman spectra of the C–H symmetric stretch region of encapsulated CH_4_ and C_2_H_6_ have been used to identify the types of crystal structures and guest molecules.^16,17^ Raman spectra of hydrocarbon hydrates have shown that the C–H stretching frequencies of hydrocarbon molecules in large cages are generally lower than those of molecules in small cages.^11,14,15^ Subramanian and Sloan rationalised this observation in terms of the guest–host intermolecular interactions using the loose cage–tight cage (LCTC) model as an explanation for matrix-isolation IR experiments.^15,18^ The variation in the C–H stretching frequencies of various hydrocarbons with varying types of water cages has been computed by quantum chemical computations.^19^ Investigating the C–H stretching frequencies of guest hydrocarbons in water cages is important for understanding the fundamental properties of gas hydrates. In addition, the guest–host interactions in gas hydrates play a role in the thermal expansion ratio of different types of guests and crystallographic structures^20^ and in the expression of a unique phenomenon known as self-preservation.^21^

In earlier studies, the temperature dependence of the C–H symmetric stretching frequencies of encapsulated CH_4_ as a guest molecule were investigated using Raman spectroscopy.^22–24^ In the case of sI CH_4_ hydrate, the thermal variation in the frequencies of CH_4_ in larger cages (sI 5^12^6^2^ cages) is greater than that of frequencies of CH_4_ in smaller cages (sI 5^12^ cages).^24^ In another earlier study, the neutron diffraction experiments for sI deuterated CD_4_ hydrate showed that CD_4_ in the sI 5^12^6^2^ cage distributes longitudinally within the cage at temperatures higher than 80 K, whereas CD_4_ in the 5^12^ cage distributes spherically around the center of the cage even at higher temperatures.^25^ These results suggest that the distance between the guest and host molecules in the sI 5^12^6^2^ cage is smaller than that in the sI 5^12^ cage. We proposed the idea that the difference in the thermal variations of the C–H symmetric stretching frequencies of CH_4_ in sI 5^12^ and 5^12^6^2^ cages is caused by the difference in distribution changes of guest CH_4_ under changing temperature.^24^ The trend of the variations of the C–H vibrational frequencies of the encapsulated hydrocarbon molecules in the gas hydrate under changing temperature is useful for better understanding the trends of the temperature change of the distribution of guest molecules. However, these trends for hydrocarbons encapsulated in sII hydrate have not been investigated. It has also been reported that the distribution of guest molecules differs depending on the geometry of guest molecules.^9,10,26,27^ Hence, additional investigation for the C–H vibrational frequencies of encapsulated hydrocarbon molecules in various combinations of host cages and guest molecules are expected.

In this study, we observed the variations in the Raman shift of C–H symmetric stretching vibrations for various guest hydrocarbon molecules in sI 5^12^ and 5^12^6^2^ cages and in sII 5^12^ and 5^12^6^4^ cages under changing temperatures. Gas hydrates of sI C_2_H_4_ hydrate, sII Kr + C_2_H_4_ hydrate, sI C_2_H_6_ hydrate, sI CH_4_ + C_2_H_6_ hydrate and sII CH_4_ + C_2_H_6_ hydrate were investigated. From these results, we discuss the variations in the Raman shift of the C–H symmetric stretching vibrations and the geometric dependence of the intermolecular interaction energies within the water cages of sI and sII hydrates with varying temperature.

## Experimental section

### Sample preparation

To synthesise gas hydrate samples, research-grade CH_4_, C_2_H_4_ and C_2_H_6_ (purities of 99.99, 99.9 and 99.99%, respectively; Takachiho Chemical Industry, Japan) and Kr (99.9% purity; Air Liquide Japan Ltd.) were used as the guest gases. These gas hydrate samples were formed from fine ice powder at 273.2 K and high-pressure conditions. For C_2_H_4_ hydrate and C_2_H_6_ hydrate, 1.2 MPa of C_2_H_4_ and C_2_H_6_ gas pressure was applied. The Kr + C_2_H_4_ hydrate was prepared from a gas mixture containing 9 mol% C_2_H_4_ and 91 mol% Kr at 1.6 MPa. sI CH_4_ + C_2_H_6_ hydrates were prepared from a gas mixture containing 70 mol% C_2_H_6_ and 30 mol% CH_4_ at 2.0 MPa. sII CH_4_ + C_2_H_6_ hydrates were prepared from a gas mixture containing 15 mol% C_2_H_6_ and 85 mol% CH_4_ at 2.0 MPa.

Fine ice powder (1.0 g) was prepared for preparing high-purity hydrate sample and was loaded into a high-pressure cell (internal volume: ∼30 mL), which was precooled in a freezer at 253 K. After loading at 253 K, the high-pressure cell was cooled to below 90 K, and pure CH_4_, C_2_H_4_, C_2_H_6_ or Kr gas was slowly introduced into the cell. The high-pressure cell was then transferred into a water bath kept at 273.2 K for hydrate formation. As the hydrates formed, the pressure decreased. When the pressure stabilised more than 12 hours later, the cell was cooled below 90 K, and the sample was retrieved from the cell.

### Raman spectroscopy

A Raman spectrometer (Jasco Corporation, RMP-210) equipped with a 532 nm excitation source (100 mW), a single holographic diffraction grating (1800 grooves per mm) and a CCD detector were used. The spectrum pixel resolution, which is the sampling interval of the spectrum, was 0.9 cm^−1^ per pixel in the range of 2500–3100 cm^−1^. The wave number was calibrated using atomic emission lines from a neon lamp. The Raman spectra for the C–H symmetric stretch region (2500–3100 cm^−1^) of the encapsulated hydrocarbon molecules in the gas hydrate water cages were obtained at ambient pressure within a temperature range of 93–183 K at 15 K intervals. The measured temperature was confirmed by using a thermocouple (Type T, 01-T, Ninomiya Electric Wire Co. Ltd., Japan). The calibrated thermocouple had an accuracy within 0.1 K. The peak positions could be rigorously analysed by fitting the data to a mixed Gaussian–Lorentzian function, which allowed us to obtain a high positional accuracy. We measured the C–H symmetric stretch of the sI C_2_H_6_ hydrate 18 times at 123 K at the same sample position. From these measurements, the standard deviation of the peak positions was found to be approximately 0.1 cm^−1^.

### Powder X-ray diffraction (PXRD)

Temperature-dependent PXRD measurements were performed using an X-ray diffractometer (40 kV, 40 mA; Rigaku model Ultima-III) with parallel beam optics and a low-temperature chamber. Finely powdered hydrate samples were mounted on a PXRD sample holder made of 2.5 mm thick Cu at a temperature of around 100 K. Each measurement was performed in a *θ*/2*θ* step scan mode with a step width of 0.02° using Cu Kα radiation (*λ* = 1.541 Å).

### Gas chromatography

Molecular compositions of CH_4_ and C_2_H_6_ in gas hydrate samples were determined using a gas chromatograph (Shimadzu Corporation, GC-2014) equipped with a packed column (Shimadzu Corporation, Sunpak-S), along with a thermal conductivity detector and fame ionisation detector.

## Results and discussion

**Fig. 1:**
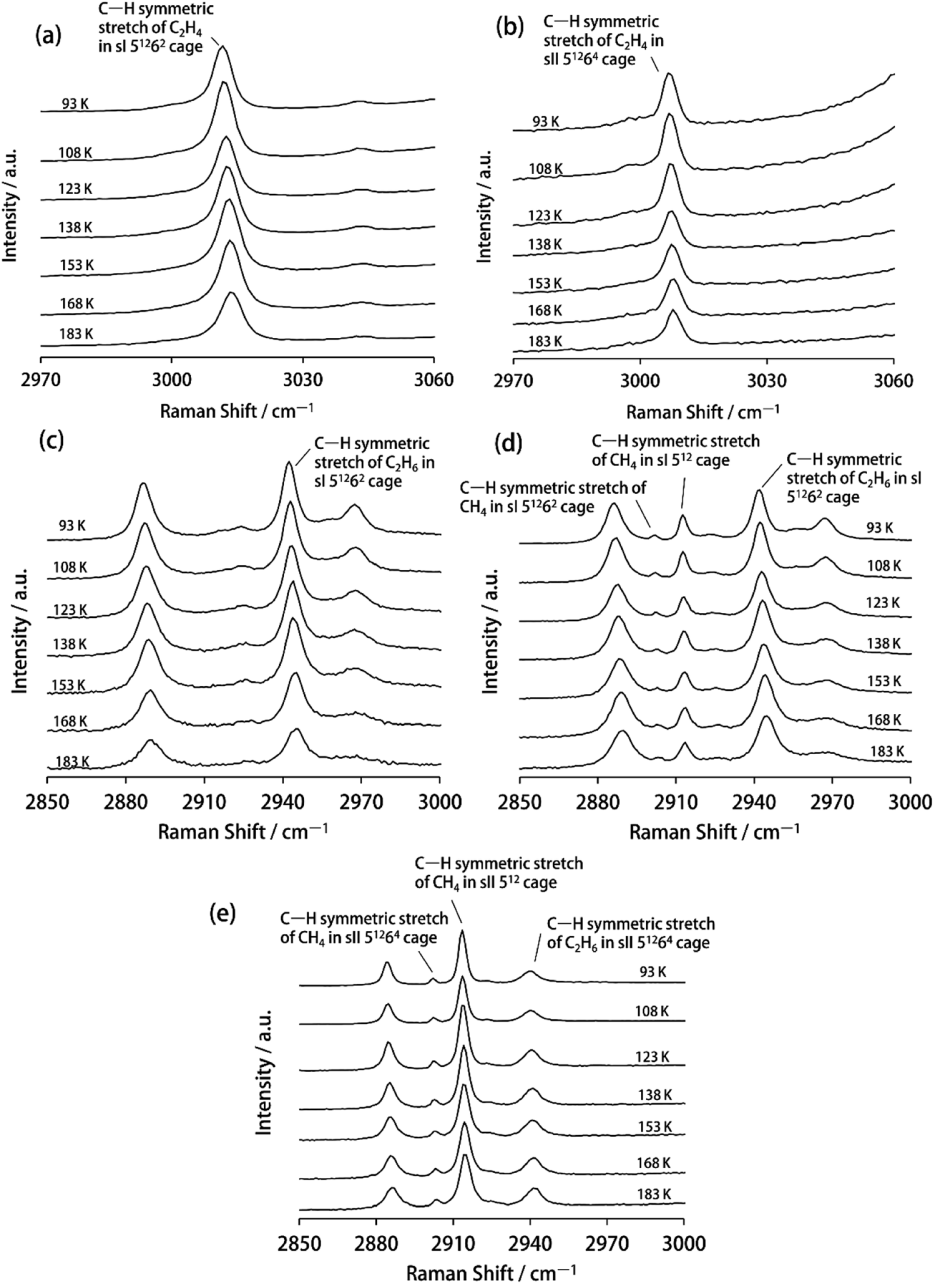
Raman spectra of the C–H stretching region of (a) sI C_2_H_4_ hydrate, (b) sII Kr + C_2_H_4_ hydrate, (c) sI C_2_H_6_ hydrate, (d) sI CH_4_ + C_2_H_6_ hydrate (*y*C_2_H_6_: 79.7%) and (e) sII CH_4_ + C_2_H_6_ hydrate (*y*C_2_H_6_: 35.3%) at a temperature range of 93–183 K.

Fig. 1 depicts the Raman spectra of the C–H stretching region of sI C_2_H_4_ hydrate, sII Kr + C_2_H_4_ hydrate, sI C_2_H_6_ hydrate, sI CH_4_ + C_2_H_6_ hydrate and sII CH_4_ + C_2_H_6_ hydrate at a temperature range of 93–183 K. We confirmed the crystal structures of sI C_2_H_4_ hydrate and sII Kr + C_2_H_4_ hydrate and their lattice constants by the PXRD method (Fig. S1 and S2†).

The Raman spectra of the C–H symmetric stretch of encapsulated CH_4_ in sI CH_4_ + C_2_H_6_ hydrate and sII CH_4_ + C_2_H_6_ hydrate were observed at 2912.7 cm^−1^ (in sI 5^12^ cages) and 2901.6 cm^−1^ (in sI 5^12^6^2^ cages); 2913.5 cm^−1^ (in sII 5^12^ cages) and 2902.2 cm^−1^ (in sII 5^12^6^4^ cages) at 93 K. Here, each *y*C_2_H_6_ (bulk guest composition of C_2_H_6_) of sI and sII CH_4_ + C_2_H_6_ hydrates were 79.7% and 35.3%, respectively.

**Fig. 2:**
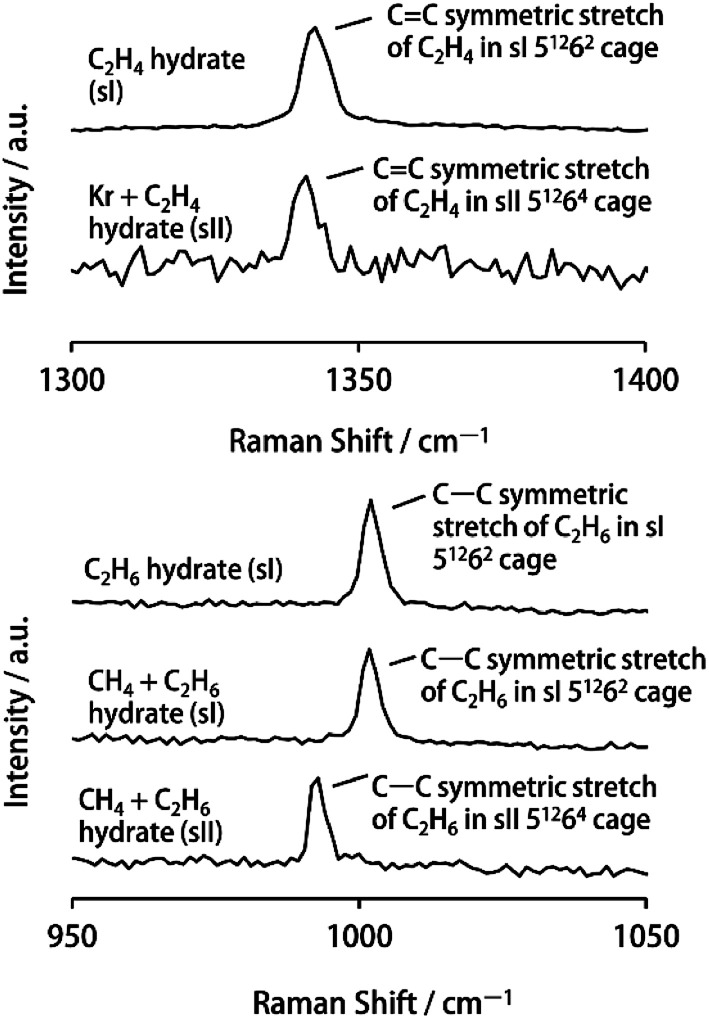
Raman spectra of C–C stretching region of sI C_2_H_4_ hydrate, sII Kr + C_2_H_4_ hydrate, sI C_2_H_6_ hydrate, sI CH_4_ + C_2_H_6_ hydrate (*y*C_2_H_6_: 79.7%) and sII CH_4_ + C_2_H_6_ hydrate (*y*C_2_H_6_: 35.3%) at 93 K.

For sI C_2_H_4_ hydrate, the Raman spectrum of the C–H symmetric stretch of encapsulated C_2_H_4_ in sI 5^12^6^2^ cages were observed at 3011.3 cm^−1^ at 93 K. This result was consistent with an earlier study.^7^ The Raman shift of the C–H symmetric stretch of encapsulated C_2_H_4_ in larger sII 5^12^6^4^ cages (3007.0 cm^−1^ at 93 K) was red-shifted relative to that of the encapsulated C_2_H_4_ in smaller sI 5^12^6^2^ cages. Furthermore, the Raman shift of the C=C symmetric stretch of the encapsulated C_2_H_4_ in sII 5^12^6^4^ cages of sII Kr + C_2_H_4_ hydrate (1340.7 ± 0.4 cm^−1^ at 93 K) was red-shifted relative to that of sI 5^12^6^2^ cages of sI C_2_H_4_ hydrate (1342.6 ± 0.4 cm^−1^ at 93 K) (Fig. 2). These results agree with the LCTC model. This is also the first report of the Raman spectrum of C_2_H_4_ encapsulated in sII hydrate.

In the case of sI C_2_H_6_ hydrate, sI CH_4_ + C_2_H_6_ hydrate and sII CH_4_ + C_2_H_6_ hydrate, the Raman spectrum of the C–H symmetric stretch of encapsulated C_2_H_6_ in the water cages of gas hydrate were observed at 2942.1 cm^−1^, 2941.9 cm^−1^ and 2940.1 cm^−1^ at 93 K, respectively. The attribution of this vibrational mode was based on the previous literature.^28^ Furthermore, structures of sI C_2_H_6_ hydrate, sI CH_4_ + C_2_H_6_ hydrate and sII CH_4_ + C_2_H_6_ hydrate samples were confirmed from the Raman spectra of the C–C symmetric stretch of encapsulated C_2_H_6_ in each gas hydrate (1002.2 ± 0.4 cm^−1^, 1001.9 ± 0.4 cm^−1^ and 992.4 ± 0.4 cm^−1^, respectively, at 93 K; see Fig. 2).^11^

**Fig. 3:**
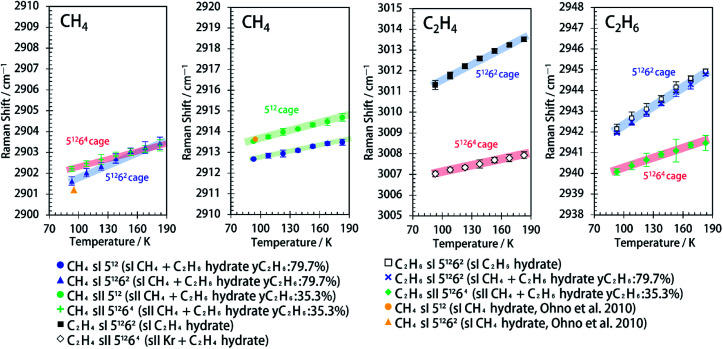
Effect of temperature on Raman shifts of C–H symmetric stretch of encapsulated CH_4_, C_2_H_4_ and C_2_H_6_ in various water cages of sI and sII hydrates.^29^

Here, the notation Δ*v*/Δ*T* refers to the temperature-dependent slope of the Raman shifts of the C–H symmetric stretching vibrations in guest hydrocarbon molecules encapsulated in the gas hydrate water cages. Fig. 3 shows Δ*v*/Δ*T* of the encapsulated CH_4_, C_2_H_4_ and C_2_H_6_ in water cages of various gas hydrates in a temperature range of 93–183 K. In the case of CH_4_, Δ*v*/Δ*T* of encapsulated CH_4_ in sI 5^12^6^2^ cages (sI CH_4_ + C_2_H_6_ hydrate) were greater than that of encapsulated CH_4_ in sII 5^12^ and 5^12^6^4^ cages. We observed that Δ*v*/Δ*T* of CH_4_ in the larger sI 5^12^6^2^ cages was greater than that of CH_4_ in 5^12^ cages of sI hydrates.^24^ Our measurements for the encapsulated CH_4_ in the sI CH_4_ + C_2_H_6_ hydrate agree with this trend. In the case of C_2_H_4_, Δ*v*/Δ*T* of C_2_H_4_ in sI 5^12^6^2^ cages (sI C_2_H_4_ hydrate) was greater than that of C_2_H_4_ in sII 5^12^6^4^ cages (sII Kr + C_2_H_4_ hydrate). Furthermore, in the case of C_2_H_6_, Δ*v*/Δ*T* of C_2_H_6_ in sI 5^12^6^2^ cages (sI C_2_H_6_ hydrate and sI CH_4_ + C_2_H_6_ hydrate) was greater than that of C_2_H_6_ in sII 5^12^6^4^ cages (sII CH_4_ + C_2_H_6_ hydrate). For CH_4_, C_2_H_4_ and C_2_H_6_ as guest molecules, Δ*v*/Δ*T* for molecules in the sI 5^12^6^2^ cages were greater than that for molecules in sII 5^12^6^4^ cages. Moreover, we found that Δ*v*/Δ*T* of CH_4_ in sII 5^12^ cages was almost the same as that of CH_4_ in sII 5^12^6^4^ cages. Specific values of Δ*v*/Δ*T* from Fig. 3 are summarised in Table 1.

**Table 1:** Raman shifts of C–H symmetric stretch of CH_4_ in sI 5^12^, 5^12^6^2^, sII 5^12^ and 5^12^6^4^ cages; C_2_H_4_ and C_2_H_6_ in sI 5^12^6^2^ and sII 5^12^6^4^ cages; and their variations with temperature changes. The errors are the standard deviations of nine measurements at different sample positions

Guest molecule	Cage	Structure	Hydrate	Raman shift at 93 K [cm^−1^]	Slope of Raman shift between 93 K and 183 K (Δ*v*/Δ*T*) [10^−2^ cm^−1^/K]
CH_4_	5^12^	sI	CH_4_ + C_2_H_6_ (*y*C_2_H_6_: 79.7%)	2912.7 ± 0.1	+0.9 ± 0.1
	5^12^6^2^	sI	CH_4_ + C_2_H_6_ (*y*C_2_H_6_: 79.7%)	2901.6 ± 0.1	+2.0 ± 0.3
	5^12^	sII	CH_4_ + C_2_H_6_ (*y*C_2_H_6_: 35.3%)	2913.5 ± 0.1	+1.3 ± 0.2
	5^12^6^4^	sII	CH_4_ + C_2_H_6_ (*y*C_2_H_6_: 35.3%)	2902.2 ± 0.1	+1.2 ± 0.1
C_2_H_4_	5^12^6^2^	sI	C_2_H_4_	3011.3 ± 0.1	+2.4 ± 0.1
	5^12^6^4^	sII	Kr + C_2_H_4_	3007.0 ± 0.1	+1.0 ± 0.2
C_2_H_6_	5^12^6^2^	sI	C_2_H_6_	2942.1 ± 0.2	+3.1 ± 0.1
	5^12^6^2^	sI	CH_4_ + C_2_H_6_ (*y*C_2_H_6_: 79.7%)	2941.9 ± 0.1	+3.2 ± 0.1
	5^12^6^4^	sII	CH_4_ + C_2_H_6_ (*y*C_2_H_6_: 35.3%)	2940.1 ± 0.1	+1.6 ± 0.1

The Raman spectra of sI and sII CH_4_ + C_2_H_6_ hydrates having different cage occupancies of large cages were obtained for verification of the effect of cage occupancies of large cages on Δ*v*/Δ*T*. The cage occupancies of large cages of sI and sII CH_4_ + C_2_H_6_ hydrates were estimated from *y*C_2_H_6_ (see Table S1†).^30^ The Raman spectra of sI and sII CH_4_ + C_2_H_6_ hydrates which have different guest composition are shown in Fig. 1 and S3,† and these Δ*v*/Δ*T* are compared in Fig. S4.† We observed the consistent trends of Δ*v*/Δ*T* of encapsulated CH_4_ and C_2_H_6_ in sI and sII CH_4_ + C_2_H_6_ hydrates regardless of different cage occupancies of large cages: Δ*v*/Δ*T* for guest molecules in the 5^12^6^2^ cages were greater than that for molecules in 5^12^ and 5^12^6^4^ cages (see Fig. S4 and Table S1†). Although different distortion and thermal expansion in sII hydrates dependent on the guest species encapsulated in 5^12^6^4^ cages were reported,^31^ it turned out to that the effect of cage occupancies of large cages have very little impact on Δ*v*/Δ*T*.

**Fig. 4:**
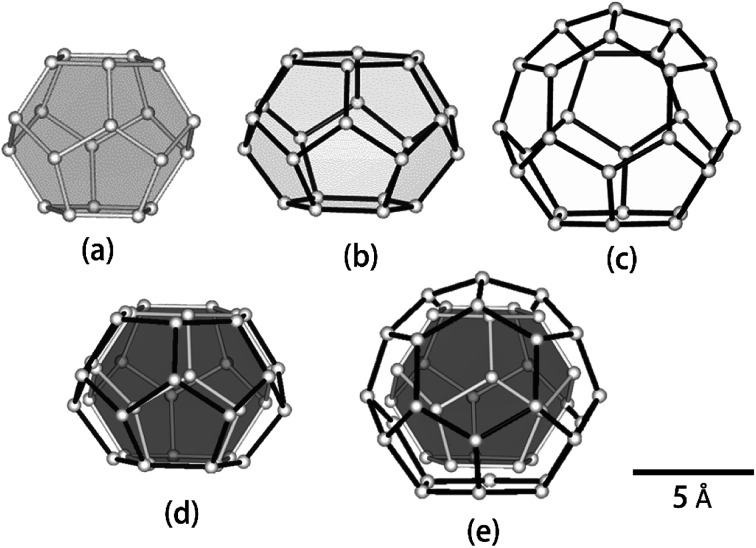
Geometry of water cages of sI and sII hydrates: (a) 5^12^ cage, (b) 5^12^6^2^ cage, (c) 5^12^6^4^ cage, (d) 5^12^ cage *vs.* 5^12^6^2^ cage and (e) 5^12^ cage *vs.* 5^12^6^4^ cage.

We compared the geometrical properties of 5^12^, 5^12^6^2^ and 5^12^6^4^ cages. sI 5^12^, sII 5^12^ and 5^12^6^4^ cages are known to be almost spherical, although they are slightly distorted, depending on the type of guest molecule. sI 5^12^6^2^ cages, however, are known to be spheroidal and extend along the equatorial plane (Fig. 4). Δ*v*/Δ*T* values of CH_4_ in both sII 5^12^ and 5^12^6^4^ cages were equivalent, although these two host water cages were the smallest and largest cages in this study, respectively (see Table 1). Δ*v*/Δ*T* of guests in the sI 5^12^6^2^ cages was greater compared with the 5^12^ cages and sII 5^12^6^4^ cages. The earlier study indicated that distributions and reorientations of guest molecules were affected by the distortion of the shapes of the host cages. For instance, C_2_H_6_ and carbon dioxide (CO_2_) molecules within the sI 5^12^6^2^ cage lie near the equatorial plane of the cages, with the long axis of the guest molecules lying in the plane.^9,10^ By contrast, the spherically extended distribution of C_3_H_8_ and C_4_H_10_ molecules in the sII 5^12^6^4^ cages obtained by the X-ray diffraction structure analysis was apparent.^26,27^ In addition, in the case of sI CD_4_ hydrate, the CD_4_ in sI 5^12^ cages showed a spherical density distribution at the centre of the 5^12^ cage at temperatures of 7.7–185 K, while CD_4_ in sI 5^12^6^2^ cages showed only a longitudinal density distribution between the two hexagonal faces of the sI 5^12^6^2^ cages.^25^ These differences in the distributions of guest molecules encapsulated in the different host water cages of gas hydrates may cause thermal vibrations in the guest molecules and variations in guest–host and guest–guest interactions due to varying temperatures.

In our previous study, we considered the effect of the size of the host cages on Δ*v*/Δ*T* and found that the slope increased with increasing host water cage volume.^24^ In this study, we revealed that Δ*v*/Δ*T* depends on the geometry as well as the volume of host water cages. For a discussion about the effect of the conformation of guest molecules on Δ*v*/Δ*T*, more studies of various combinations of guest molecules and host water cages are needed. These experimental trends of Δ*v*/Δ*T* for the various water cages of gas hydrates may advance our understanding of the fundamental properties of sI and sII hydrocarbon gas hydrates.

## Conclusions

In this work, we investigated the slopes of C–H symmetric stretching vibrational frequencies under changing temperatures (Δ*v*/Δ*T*) for various hydrocarbon molecules in 5^12^, 5^12^6^2^ and 5^12^6^4^ cages of sI and sII hydrates. Δ*v*/Δ*T* values of CH_4_, C_2_H_4_ and C_2_H_6_ molecules encapsulated in sII 5^12^6^4^ cages were smaller than those for molecules in sI 5^12^6^2^ cages. In our previous study, we suggested that Δ*v*/Δ*T* is greater with increasing volume of host water cages. In this study, we revealed that Δ*v*/Δ*T* is dependent on the geometry, as well as the volume of host water cages and guest molecules.

In the future, we intend to investigate the effects of the conformation of guest molecules on Δ*v*/Δ*T*. For a discussion about the effect of the conformation of guest molecules on Δ*v*/Δ*T*, more studies of various combinations of guest molecules and host water cages are required.

## Conflicts of interest

There are no conflicts to declare.

## Supplementary Material

RA-010-D0RA06668K-s001
