# Supplementary material for: Temperature effects on the C–H symmetric stretching vibrational frequencies of guest hydrocarbon molecules in 512, 51262 and 51264 cages of sI and sII clathrate hydrates
Source: RSC Adv. 2020 Oct 12;10(61):37582–7. doi: 10.1039/d0ra06668k (PMC9057130; doi:10.1039/d0ra06668k)
Supplement: RA-010-D0RA06668K-s001 [file RA-010-D0RA06668K-s001.pdf]

Electronic Supplementary Material (ESI) for RSC Advances.
This journal is © The Royal Society of Chemistry 2020

Supporting Information

Temperature effects on the C–H symmetric stretching vibrational frequencies of guest hydrocarbon molecules in 5^{12}, $5^{12}6^{2}$ and $5^{12}6^{4}$ cages of sI and sII clathrate hydrates

Go Fuseya and Akihiro Hachikubo*
Kitami Institute of Technology, 165, Koen-cho, Kitami 090-8507, Japan

Satoshi Takeya
National Institute of Advanced Industrial Science and Technology (AIST), Central 5, 1-1-1, Higashi, Tsukuba 305-8565, Japan

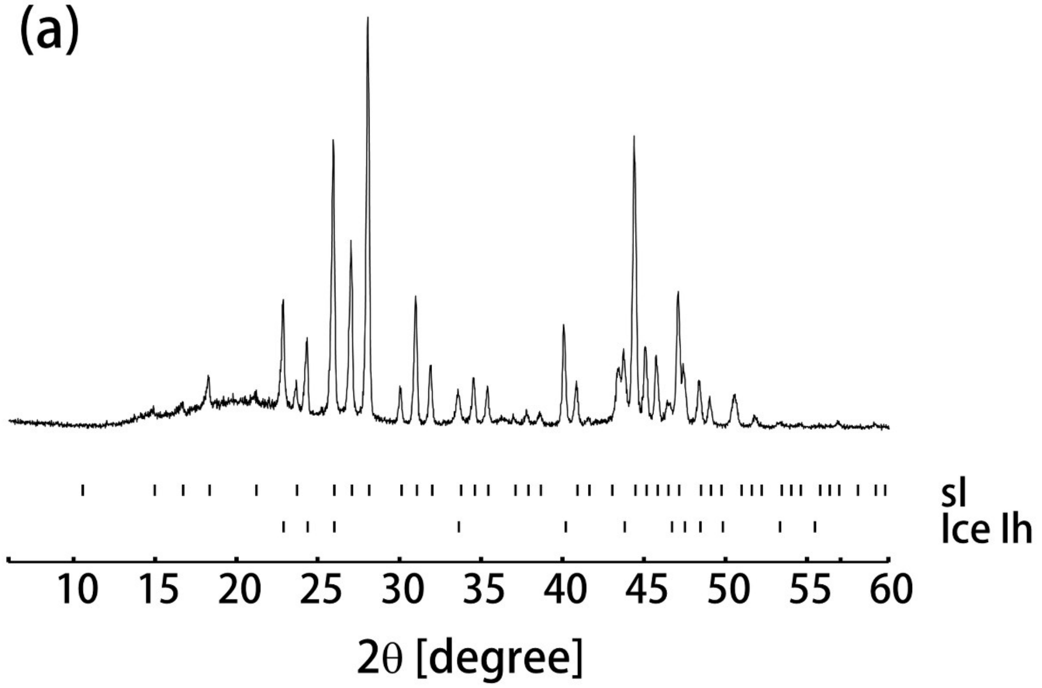

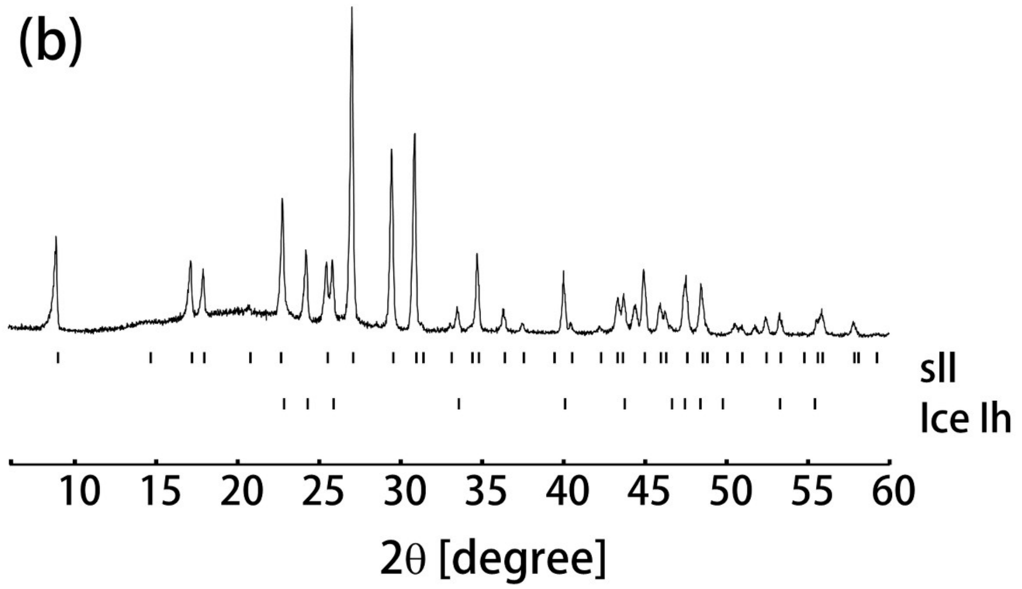

Fig. S1. X-ray diffraction patterns of the hydrate samples at 93 K: (a) sI C_2H_4 hydrate and (b) sII Kr + C_2H_4 hydrate. The curves in each pattern represent the observed intensities. The line marks below each pattern show the calculated peak positions for sI hydrate, sII hydrate and hexagonal ice.

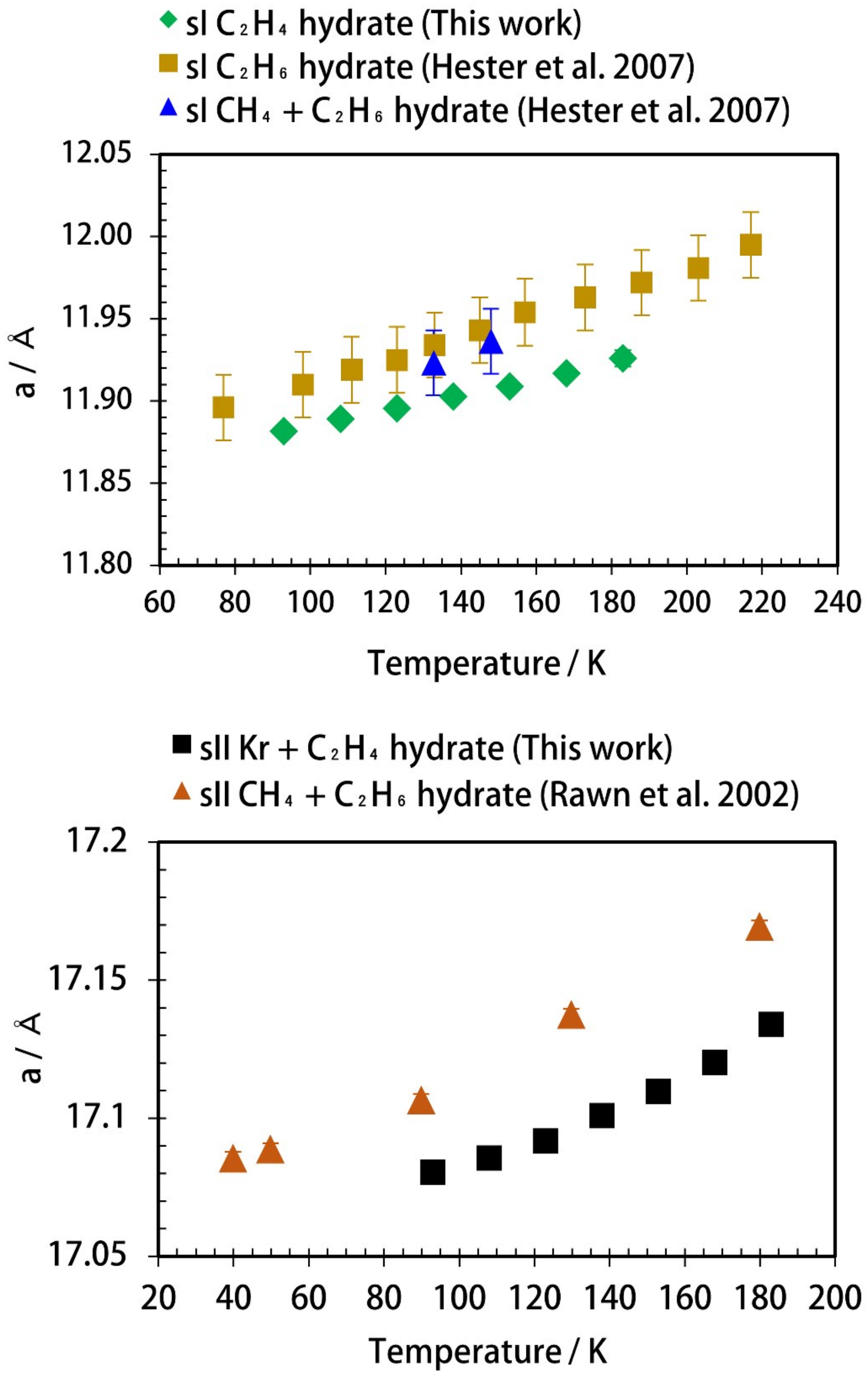

Fig. S2. Effect of temperature on unit-cell parameters of five types of sI and sII hydrates.[1,2]

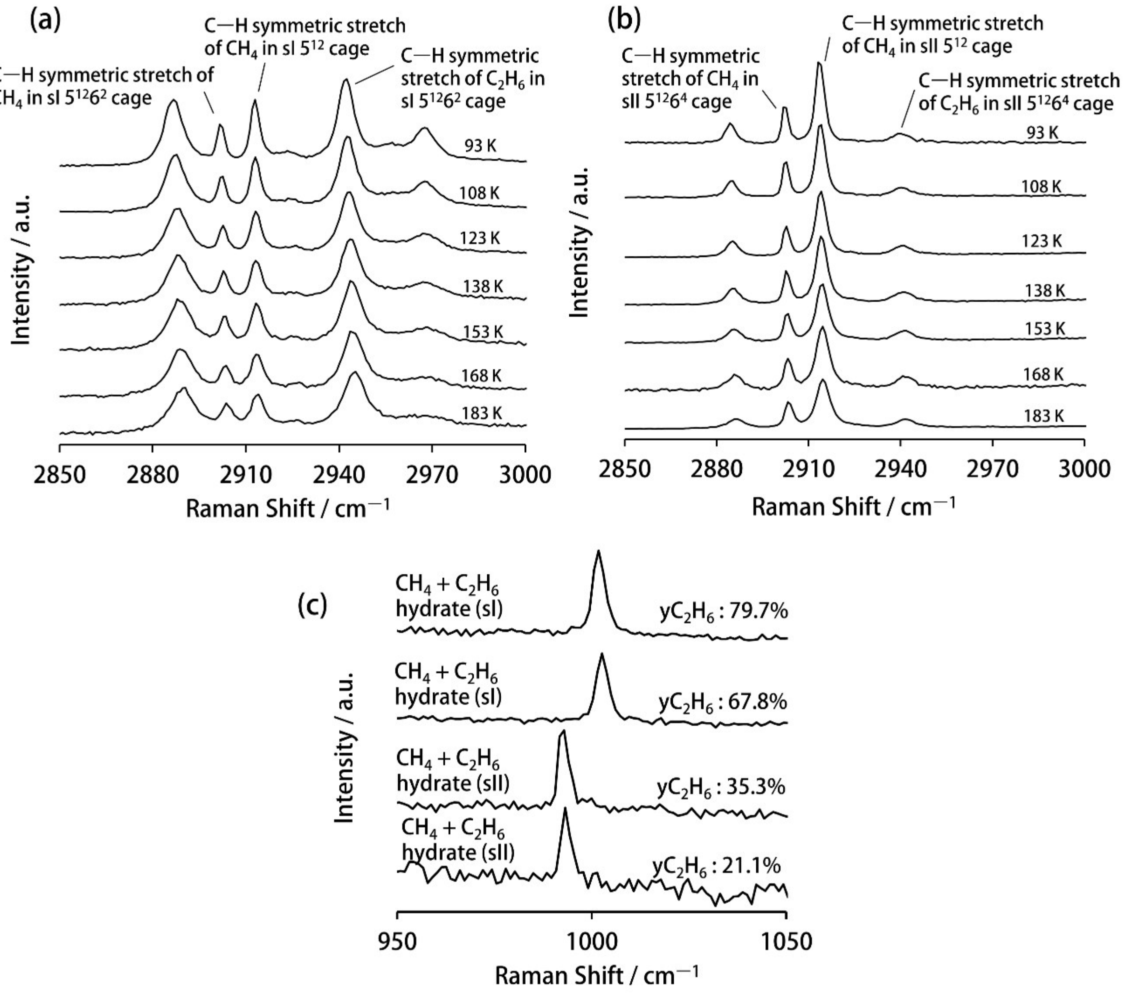

Fig. S3. Raman spectra of the C–H stretching region of (a) sI CH_4 + C_2H_6 hydrates (yC_2H_6 : 67.8%) and (b) sII CH_4 + C_2H_6 hydrates (yC_2H_6 : 21.1%) at a temperature range of 93–183 K. (c) Raman spectra of the C–C stretching region of encapsulated C_2H_6 in sI and sII CH_4 + C_2H_6 hydrates at 93 K.

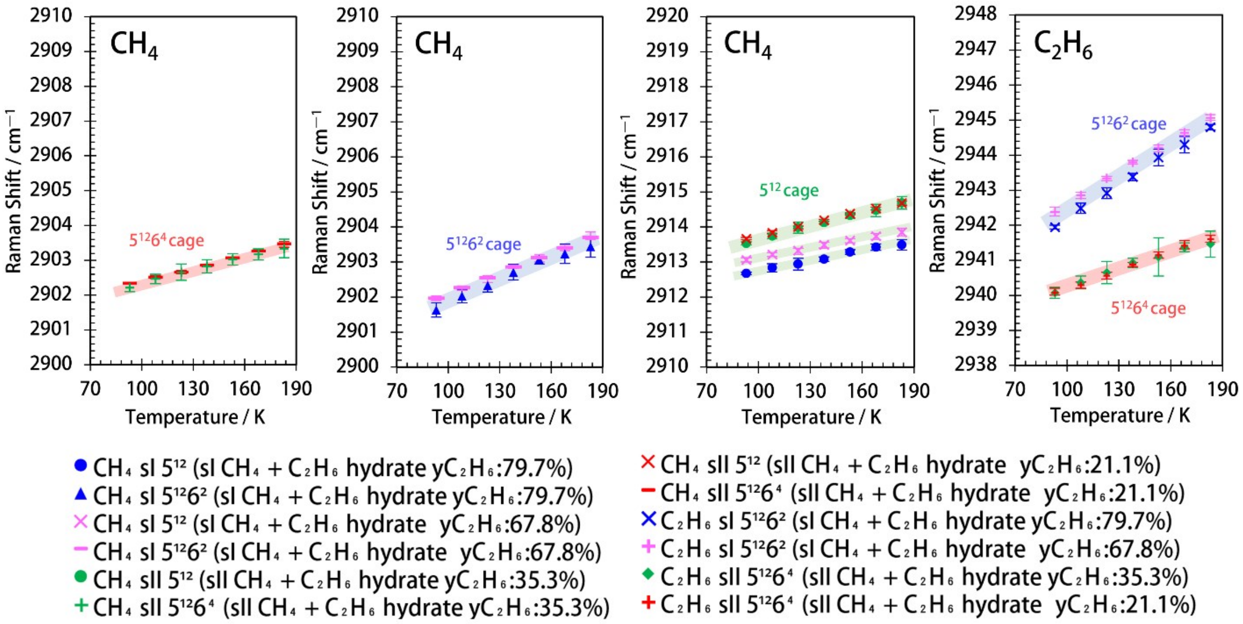

Fig. S4. Effect of temperature on Raman shifts of C–H symmetric stretch of encapsulated CH_4 and C_2H_6 in sI and sII CH_4 + C_2H_6 hydrates which have different guest composition.

Table S1 Raman shifts of C−H symmetric stretch of CH_4 and C_2H_6 in sI and sII CH_4 + C_2H_6 hydrates which have different guest composition and their variations with temperature changes. The errors are the standard deviations of nine measurements at different sample positions.

guest molecule	cage	structure	bulk guest composition of C_2H_6 [%]	estimated cage occupancy ratio of CH_4 in large cages[3]	estimated cage occupancy ratio of C_2H_6 in large cages[3]	Raman shift at 93 K [cm^{-1}]	slope of Raman shift between 93 K and 183 K ($\Delta\nu/\Delta T$) [10^{-2} cm^{-1}/K]
CH_4	5^{12}	sI	79.7	0.04	0.94	2912.7 ± 0.1	+0.9 ± 0.1
	$5^{12}6^{2}$	sI	79.7	0.04	0.94	2901.6 ± 0.1	+2.0 ± 0.3
	5^{12}	sI	67.8	0.12	0.86	2913.1 ± 0.1	+0.9 ± 0.1
	$5^{12}6^{2}$	sI	67.8	0.12	0.86	2902.0 ± 0.1	+1.9 ± 0.1
	5^{12}	sII	35.3	0.14	0.83	2913.5 ± 0.1	+1.3 ± 0.2
	$5^{12}6^{4}$	sII	35.3	0.14	0.83	2902.2 ± 0.1	+1.2 ± 0.1
	5^{12}	sII	21.1	0.40	0.56	2913.7 ± 0.1	+1.2 ± 0.1
	$5^{12}6^{4}$	sII	21.1	0.40	0.56	2902.3 ± 0.1	+1.3 ± 0.1
C_2H_6	$5^{12}6^{2}$	sI	79.7	0.04	0.94	2941.9 ± 0.1	+3.2 ± 0.1
	$5^{12}6^{2}$	sI	67.8	0.12	0.86	2942.4 ± 0.1	+3.0 ± 0.1
	$5^{12}6^{4}$	sII	35.3	0.14	0.83	2940.1 ± 0.1	+1.6 ± 0.1
	$5^{12}6^{4}$	sII	21.1	0.40	0.56	2940.1 ± 0.1	+1.7 ± 0.1

References

[1] K. C. Hester, Z. Huo, A. L. Ballard, C. A. Koh, K. T. Miller and E. D. Sloan, *J. Phys. Chem. B*, 2007, **111**, 8830–8835.

[2] C. J. Rawn, A. J. Rondinone, B. C. Chakoumakos, S. L. Marshall, L. A. Stern, S. Circone, S. Kirby, C. Y. Jones, B. H. Toby, and Y. Ishii, Neutron Powder Diffraction Studies as a Function of Temperature of sII Hydrate Formed from a Methane + Ethane Gas Mixture. Proceedings of the 4th International Conference on Gas Hydrates, Yokohama, Japan, 2002.

[3] M. Kida, H. Sakagami, N. Takahashi, A. Hachikubo, H. Shoji, Y. Kamata, T. Ebinuma, H. Narita and S. Takeya, *J. Jpn. Petrol. Inst.*, 2007, **50**, 132–138.
